# A Detailed Analysis of Parameters Supporting the Engraftment and Growth of Chronic Lymphocytic Leukemia Cells in Immune-Deficient Mice

**DOI:** 10.3389/fimmu.2021.627020

**Published:** 2021-03-09

**Authors:** Piers E. M. Patten, Gerardo Ferrer, Shih-Shih Chen, Jonathan E. Kolitz, Kanti R. Rai, Steven L. Allen, Jacqueline C. Barrientos, Nikolaos Ioannou, Alan G. Ramsay, Nicholas Chiorazzi

**Affiliations:** ^1^ Institute of Molecular Medicine, Karches Center for Oncology Research, The Feinstein Institutes for Medical Research, Northwell Health, Manhasset, NY, United States; ^2^ Faculty of Life Sciences & Medicine, School of Cancer & Pharmaceutical Sciences, Comprehensive Cancer Centre, Institute of Haematology, King’s College London, London, United Kingdom; ^3^ Department of Medicine, Zucker School of Medicine at Hofstra/Northwell, Hempstead, NY, United States; ^4^ Department of Molecular Medicine, Zucker School of Medicine at Hofstra/Northwell, Hempstead, NY, United States

**Keywords:** chronic lymphocytic leukemia, patient-derived xenograft, engraftment, growth, T cells, B cells

## Abstract

Patient-derived xenograft models of chronic lymphocytic leukemia (CLL) can be created using highly immunodeficient animals, allowing analysis of primary tumor cells in an *in vivo* setting. However, unlike many other tumors, CLL B lymphocytes do not reproducibly grow in xenografts without manipulation, proliferating only when there is concomitant expansion of T cells. Here we show that *in vitro* pre-activation of CLL-derived T lymphocytes allows for a reliable and robust system for primary CLL cell growth within a fully autologous system that uses small numbers of cells and does not require pre-conditioning. In this system, growth of normal T and leukemic B cells follows four distinct temporal phases, each with characteristic blood and tissue findings. Phase 1 constitutes a period during which resting CLL B cells predominate, with cells aggregating at perivascular areas most often in the spleen. In Phase 2, T cells expand and provide T-cell help to promote B-cell division and expansion. Growth of CLL B and T cells persists in Phase 3, although some leukemic B cells undergo differentiation to more mature B-lineage cells (plasmablasts and plasma cells). By Phase 4, CLL B cells are for the most part lost with only T cells remaining. The required B-T cell interactions are not dependent on other human hematopoietic cells nor on murine macrophages or follicular dendritic cells, which appear to be relatively excluded from the perivascular lymphoid aggregates. Notably, the growth kinetics and degree of anatomic localization of CLL B and T cells is significantly influenced by intravenous versus intraperitoneal administration. Importantly, B cells delivered intraperitoneally either remain within the peritoneal cavity in a quiescent state, despite the presence of dividing T cells, or migrate to lymphoid tissues where they actively divide; this dichotomy mimics the human condition in that cells in primary lymphoid tissues and the blood are predominately resting, whereas those in secondary lymphoid tissues proliferate. Finally, the utility of this approach is illustrated by documenting the effects of a bispecific antibody reactive with B and T cells. Collectively, this model represents a powerful tool to evaluate CLL biology and novel therapeutics *in vivo*.

## Introduction

Patient-derived xenograft (PDX) models of chronic lymphocytic leukemia (CLL) can help analyze the biology of primary leukemic B and T cells in an *in vivo* setting (1–7). However, creating successful xenografts requires surmounting several inherent barriers, the most significant being the transfer and growth for a relatively long period of time of cells of one species into recipients of another. This difficulty has been obviated to a great degree by using severely immune-deficient mice lacking mature T cells, B cells and NK cells (“alymphoid mice”). A commonly used recipient strain of such mice is the NOD-*scid* IL2Rgamma^null^ animal, referred to as the “NSG” mouse. Another major barrier to successful xenografting is pulling together sufficient environmental cues, from the donor and/or the host, to allow not only the survival but also the growth of the transferred cell population.

We previously used NSG animals to develop a PDX model in which transfer of CLL peripheral blood mononuclear cells (PBMCs) along with allogeneic antigen-presenting cells (APCs) led to *in vivo* CLL-derived T-cell activation that promoted survival and growth of the leukemic cells (4). In this model, the presence of activated T cells was essential for successful CLL B-cell proliferation since CLL B-cell growth was only found when concomitant expansion of autologous T cells was observed. Moreover, elimination of T lymphocytes, in particular CD4^+^ cells, at the initiation of engraftment prevented growth of the leukemic B cells (4).

This approach has advantages and disadvantages. Positive aspects include the simplicity of the technique, the relatively small numbers of CLL B and T cells needed to achieve a productive outcome, and the ready promotion of CLL-cell growth *in vivo.* The major negative feature is the dependence on T-cell activation taking place *in vivo* as a consequence of the donor T cells recognizing the foreign histocompatibility antigens of the provoking, co-administered human APCs. Although effective in most instances, the level of histocompatibility difference between the antigen-presenting cell of the normal donor and the T lymphocytes from the CLL-cell donor is rarely, if ever known. Therefore, the extent and degree of CLL T-cell activation that can occur in the recipient animals differs and is not readily predictable and quantifiable in advance of cell transfer. Consequently, the extent of T-cell help provided for leukemic B-cell proliferation cannot be foretold and controlled to make robust comparisons between experiments involving a diverse set of donors.

Here we address the hypothesis that *in vitro* pre-activation of CLL-derived T lymphocytes prior to xenografting with autologous CLL cells provides a more reliable and constant source, on a per cell basis, of T-cell help for the growth of leukemic B cells in NSG recipients. We show that this approach leads to reproducible growth of primary CLL cells within a fully autologous system using limited numbers of leukemic cells from a broad range of patients. We also detail extensive studies of CLL-cell proliferation and how these relate to CLL-derived human T cells, murine hematopoietic and non-hematopoietic cells, route of administration, and the need to pre-condition recipients. An example of the utility of these improvements in testing the efficacy of a novel therapeutic is provided.

## Materials and Methods

### Chronic Lymphocytic Leukemia Patient Samples and Characterization

In accordance with the Declaration of Helsinki and as approved by the Institutional Review Board of Northwell Health, after obtaining informed consent, blood was collected from 19 CLL patients for whom clinical information, laboratory data, and *IGHV-IGHD-IGHJ* DNA sequences [**Table 1** and (8)] were available. PBMCs were separated by density gradient centrifugation using Ficoll Paque Plus (GE Healthcare Life Sciences) and cryopreserved until use in RPMI-1640 medium (Invitrogen) supplemented with 10% heat-inactivated fetal bovine serum (FBS, Atlanta Biologicals).

**Table 1:** Characteristics of the patients used in this study.

Patient ID #	Ig Isotype	*IGHV*	*IGHV* mutation status	Cytogenetics							
				6q23.3	11q13	11q22.3	12 centromere	13q14.3	13q34	14q32	17p13
0515	IgM	4-39	U	N	N	N	N	A	N	N	N
0545	IgM	3-30	M	N	N	N	N	N	N	N	N
0854	IgM	1-03	M	ND	ND	N	N	A	N	ND	N
1083	IgM	4-b	U	N	N	A	N	A	N	N	N
1122	IgM	3-09	U	ND	N	N	A	N	N	N	N
1164	IgM	4-34	M	N	N	N	N	N	N	N	N
1279	IgM	1-02	U	ND	ND	N	N	A	N	N	N
1301	IgM	4-31	U	N	N	N	A	N	N	N	N
1429	IgG	3-48	M	N	ND	N	N	A	N	ND	N
1435	IgM	1-08	M	N	N	N	N	N	N	N	N
1463	IgM	3-21	M	N	N	N	N	A	N	N	N
1493	ND	33-03	M	N	N	N	N	A	N	N	N
1523	IgM	3-48	U	ND	N	N	N	A	N	N	N
1539	IgM	3-30	M	ND	ND	N	N	A	ND	ND	A
1552	ND	1-18	M	N	N	N	N	A	N	N	N
1623	IgM	2-70	M	ND	ND	ND	ND	ND	ND	ND	ND
1925	ND	3-11	U	N	N	A	N	A	N	N	N
2030	ND	3-30	U	A	A	N	N	A	A	A	N
2156	IgM	V5-51	U	N	N	A	N	A	N	N	N

IGHV mutation status is mutated (M) if the expressed IGHV sequence is >2% different from germline; otherwise it is considered IGHV-unmutated (U).

Cytogenetics was assigned as abnormal (A) or normal (N) by FISH.

ND, Not done.

### Xenogeneic Transplantation

Four to 8 week old NOD-scid IL2Rgamma^null^ (“NSG™”, Jackson Laboratory) mice were used as xenograft recipients for cryopreserved CLL cells from only a single donor. If a sample had reduced viability, it was centrifuged through a Ficoll gradient to remove excess dead cells. For those experiments where extent of cell division was determined *in vivo*, cells were incubated 10 minutes at 37°C with CFSE (2.5μM; Invitrogen) and washed with cold culture medium just before transfer.

For those experiment requiring activated autologous T lymphocytes, CD3^+^ cells from a single CLL patient were enriched from PBMCs using anti-CD3 microbeads (Miltenyi Biotec) by following the manufacturer’s recommended protocol. Then, 1 × 10^6^ CD3^+^cells per mL were cultured for 3–14 days with 25 µl of human T-Activator CD3/CD28 Dynabeads (Invitrogen) and 30 units of IL-2 (R&D) per mL of cells in culture medium (RPMI-1640 supplemented with 10% heat-inactivated FBS and antibiotics (GE Healthcare Life Sciences)) at 37°C, 95% humidity and 5% of CO_2_. Cultures were maintained at 1 × 10^6^ cells/mL with fresh culture medium containing IL-2. In those instances when cells were cultured for > 7 days, beads were removed using a magnet, and the CD3^+^cells were re-exposed to new anti-CD3/28 beads and fresh culture medium. At the end of the T-cell activation period, autologous CLL PBMCs from the same patient (all experiments) were thawed and evaluated by trypan blue (Thermo Fischer Scientific).

Then 20 × 10^6^ viable PBMCs were mixed with CD3^+^ (at a 1:40 ratio of CD3^+^ cells: PBMCs) or without CD3^+^ activated cells and injected.

Each individual NSG mouse then received 20 million live cells resuspended in 50-100 ml PBS transferred either intravenously (iv) *via* the retro-orbital plexus or intraperitoneally (ip) by percutaneous injection. Additionally, in a subset of studies, mice received 25 mg/kg busulfan ip for microenvironmental preconditioning 24 h prior to xenografting. Following injection, mice were bled and sacrificed as described for each experiment. Human cells and murine sera were evaluated as indicated below.

### Assessment of Blood, Bone Marrow, and Splenic Tissue At Euthanasia

At euthanasia, spleens were bisected in order to prepare single cell suspensions for flow cytometry (FC) studies and tissue blocks were made for microscopy studies. Antibodies used for FC studies are listed in **Table S1** and primary antibodies for microscopy studies in **Table S2**. All FC data were acquired with a BD LSRII flow cytometer (Becton Dickinson Immunocytometry Systems) and analyzed by FlowJo V10.6.2 software (TreeStar). Absolute numbers of human cells were calculated using the cell count from the single cell suspension obtained at the time of processing, and the percentage was identified by FC. The VECTASTAIN^®^ ABC system (Vector Laboratories) was used to visualize primary antibodies for light microscopy studies. Primary antibodies for immunofluorescent microscopy were visualized with affinity purified donkey IgG antibodies (Jackson Immunoresearch). Confocal microscopy was performed using either an Olympus IX70 microscope or a Nikon A1R confocal microscope. All images obtained were edited for optimal color contrast using Adobe Creative Cloud v5.1.0.407 (Adobe Systems).

### Measurement of Secreted Human IgG

Human IgG concentrations in murine plasma were measured by ELISA. 96-well flat-bottom microplates (Corning) were coated with 100 µl/well of 5 µg/ml goat F(ab)’_2_ anti-human IgG polyclonal antibodies (pAbs) (Southern Biotech). After overnight incubation, blocking and washing, wells were incubated with 80 µl/well dilutions of samples and standards (IgG from human serum; Sigma), again washed, and then mixed with 100 µl/well of a 1:8,000 dilution of a 1:1 mixture of goat anti-human Ig kappa and anti-human Ig lambda pAbs conjugated with horseradish peroxidase (Southern Biotech). Finally, plates were incubated with TMB Sure Blue (KPL), reactions stopped with 60 µl/well of 1N HCl (Fisher Scientific), and absorption at 450 nm measured on an ELx808 absorbance microplate reader (BioTek Instruments, Inc). IgG concentrations were calculated based on standard curves using the instrument’s KCjunior (v1.22) software. Human IgG detection limit varied from 0.3 to 37.8 µg/ml.

### Assessment of Plasma IFNγ

Plasma was collected at the time of animal sacrifice and stored at -80°C until use. Levels of IFNγ were measured by cytometric bead array (BD Biosciences). Results were correlated with the sum of the numbers of human CD45^+^CD4^+^CD5^+^, CD45^+^CD8^+^CD5^+^, and CD45^+^CD4^+^CD8^+^CD5^+^ cells expressed as a ratio over the number of mCD45^+^hCD45^-^ cells obtained using identical FSC and SSC gates from FACS analysis of single cell splenic suspensions obtained at the same time point.

### Statistical Analyses

All statistical tests were performed using Prism v8 (GraphPad Software, Inc). Normality was assessed using the D’Agostino-Pearson Omnibus Test, and appropriate parametric and non-parametric analyses performed thereafter. Mann-Whitney U tests were used for analyses of CLL T- and B-cell numbers in comparison experiments using PBMCs alone, PBMCS plus activated T cells, and the different injection routes (iv versus ip). For the experiments using DART molecules, Kruskal-Wallis multiple comparison tests were performed for analysis of plasma IFNγ and IgG levels and the percent of CD5^+^CD19^+^ cells obtained from the 3 groups of animals.

## Results

### Transfer of Unmanipulated Chronic Lymphocytic Leukemia Peripheral Blood Mononuclear Cells Into NSG Mice Leads To Inefficient CLL B-Cell Growth That Is Only Substantial When Autologous T-Cell Expansion Spontaneously Occurs

**Figure 1:**
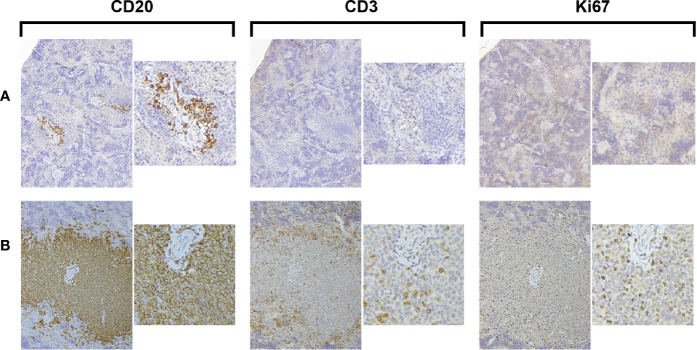
Growth of CLL B cells only occurs when there is associated expansion of autologous T cells. Representative × 10 original magnification IH images of splenic tissue obtained at euthanasia **(A)** Few CD20^+^ cells with no CD3^+^ cells or Ki67^+^ cells are present (CLL1083, representative result from 10/15 animals). **(B)** Aggregates of CD20^+^ and CD3^+^ cells with Ki67^+^ cells are apparent around blood vessels (perivascular aggregates, PVAs) (CLL1279, representative of 5/15 animals).

The transfer of solely unmanipulated primary CLL PBMCs intravenously into alymphoid mice can result in CLL recovery from the spleen after a period of 3–4 weeks (1, 5, 7). We performed such experiments transferring CLL PBMCs into unconditioned NSG animals to assess the frequency of successful xenografting, spontaneous *in vivo* T-cell activation, and growth of CLL B and T cells. In two thirds of the animals, such transfers led to the detection of none or only scanty, apparently resting CLL B cells (**Figure 1A**); in the remaining mice, CLL B-cell expansion was found (**Figure 1B**). Notably, in all those animals with CLL B-cell expansion, there was concomitant autologous T-cell growth. Moreover, evidence for cellular proliferation, based on the presence of Ki67^+^ cells, was also found only in those animals with T-cell expansion (**Figure 1B**).

Collectively, these findings confirm that primary CLL B cells grow in alymphoid mice only when there is a concurrent expansion of T cells (4). The frequency that T-cell activation spontaneously occurs and consequently leads to CLL B-cell growth when transferring unmanipulated CLL PBMCs is low (~33% in these experiments).

### Co-Transfer of Autologous T Cells, Activated Polyclonally *In Vitro*, Leads To Much Greater and More Reproducible Growth of Chronic Lymphocytic Leukemia B Cells in NSG Mice

**Figure 2:**
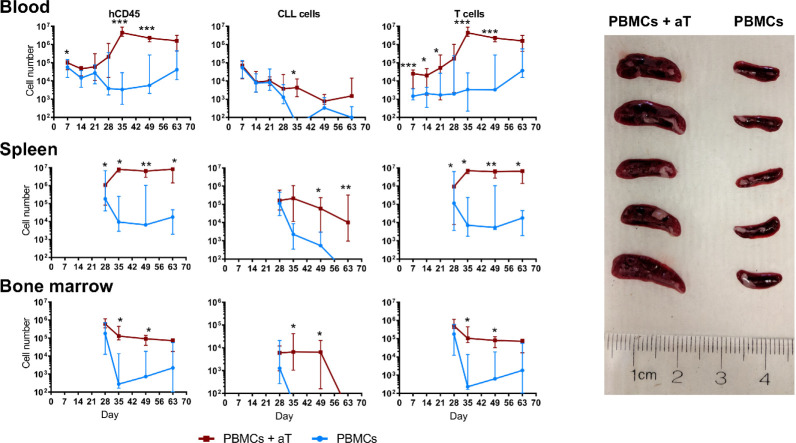
Co-injection of autologous activated T cells with CLL PBMCs leads to more effective engraftment and growth in NSG mice. Time course quantification by FC of human CD45^+^ cells, CLL B cells and autologous CLL T cells from single cell suspensions in the peripheral blood, spleen and bone marrow of 4 different patients evaluated independently. PBMCs with or without aT were injected into 5 mice per group and bled weekly up to euthanasia at week 4 (n = 40 mice); for two of the patients, additional groups of mice were injected, and these were euthanized at weeks 5, 7 and 9. Points represent the median and the interquartile range. * corresponds to Mann-Whitney U test *P* values < 0.05, **P < 0.01, and ***P < 0.001. On the right, spleens of 10 mice at week 5 post injection of 20 × 10^6^ CLL PBMCs with (Left; n = 5) and without (Right; n = 5) 0.5 × 10^6^ activated T cells (aT).

To overcome the above variability, we tested if co-transfer of a fixed numbers of pre-activated autologous T cells with CLL B cells would reproducibly lead to a greater and more prolonged proliferation of leukemic B cells than transferring PBMCs alone. To do so, we injected into NSG mice 20 × 10^6^ PBMCs alone or in combination with 0.5 × 10^6^ pre-activated T cells (1:40 T:B) from 4 CLL patients (2 *IGHV*-mutated, M-CLL cases 1493 and 1521, and 2 *IGHV*-unmutated, U-CLL cases 2030 and 2156), and recipient animals were bled weekly for 3 weeks. Finally, mice were randomly assorted into groups of 5 mice per patient sample and euthanized at weeks 4, 5, 7 and 9; blood, spleen and bone marrow (BM) samples were collected at each time point. For each group of animals, we calculated the absolute numbers of different cell types present at each site (**Figure 2**). We refer to the combination of CLL PBMCs plus *in vitro* autologous, activated T cells (aT) hereafter as “PBMCs + aT”.

Upon analyzing blood samples, human CD45-expressing cells were identified from days 7 through 63 (**Figure 2**). When focusing on CLL B cells, the numbers in the blood diminished progressively from the time of injection through week 3 for both the PBMC only and the PBMC + aT groups (**Figure 2**); this is consistent with B lymphocytes recirculating less and being more tissue resident (9, 10). However, the degree of change between the two groups differed with a lesser fall in the PBMC + aT groups than PBMCs alone. For the PBMC alone group, B cells were no longer detectable after day 56.

In contrast, T-cell counts behaved differently. At the time of the first blood sampling (day 7), the PBMC + aT group had a significantly higher number of T cells, possibly influenced by the higher number of T lymphocytes transferred initially (**Figure 2**). However, between days 14 and 35, T-cell counts between the two groups diverged appreciably due to *in vivo* expansion. This numerical superiority reached a peak at day 35 and continued to be maximally divergent until the last bleeding (day 63) (**Figure 2**).

These differences in the numbers of CD45^+^ and CLL B and T cells in the blood were mirrored temporally in the spleens (**Figure 2**) and BMs (**Figure 2**) of the two groups of animals, with day 35 being the critical point at which the groups significantly diverged. This disparity was reflected by the clear dominance in spleen sizes for the PBMC + aT group at each time that euthanasia was carried out (**Figure 2**).

Finally, it is notable that T cell numbers remained relatively constant from day 35 until the end of the experiment (day 63) (**Figure 2**). In contrast, the numbers of CLL B cells started to decline beginning at day 49. As will be addressed below, the time points at which individual patient samples reach maximal and minimal numbers of CLL B and T cells differ for specific samples.

### No Clear Advantage To Pre-Conditioning NSG Recipients For Xenografting Mature Chronic Lymphocytic Leukemia Cells

**Figure 3:**
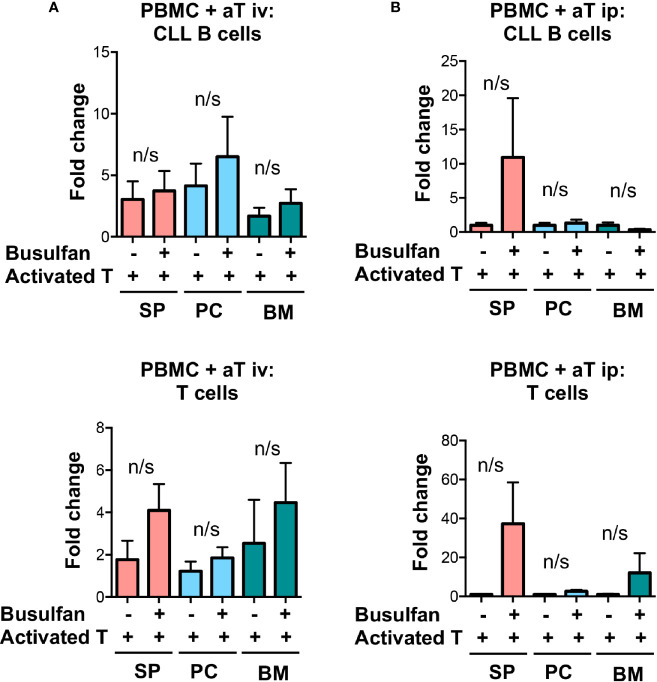
Busulfan preconditioning does not provide a clear advantage for the xenografting of primary CLL cells in the PBMC + aT model. **(A)** Five NSG mice did not or did receive 25mg/kg busulfan ip 24 h prior to xenografting. Then, 20 × 10^6^ CLL PBMCs with 0.5 × 10^6^ activated T cells (aT) were injected iv into NSG mice. Five weeks after cell injection, mice were sacrificed and single cell suspensions from blood, spleen, bone marrow (BM) and peritoneum were analyzed by flow cytometry. Busulfan did not significantly improve CLL B-cell (top) and T-cell (bottom) engraftment. Data represent a composite of experiments involving cells from 4 patients, 2 U-CLL and 2 M-CLL. **(B)** Similar busulfan preconditioning was given or not to two other sets of 5 NSG mice that received samples from 4 different patients (2 U-CLL and 2 M-CLL). Twenty-four h after, 20 × 10^6^ CLL PBMCs with 0.5 × 10^6^ activated T cells (aT) were injected ip into each recipient mouse. Although there is a trend for better engraftment of CLL B and T cells in busulfan-pretreated mice, there are no significant differences between the numbers of CLL B and T cells in any of the groups. Bar graphs represent the mean fold change (after setting the average cell counts obtained from PBMC mouse spleens as 1); S.E.M. determined by Mann-Whitney U test. n/s: no statistically significant difference.

Busulfan pre-conditioning can support the transfer of unmanipulated CLL PBMCs (5). Therefore, we tested if this type of pre-conditioning improved engraftment and growth in the PBMC + aT model. To do so, we injected busulfan ip into 50% of NSG recipients and then transferring iv, into all animals, PBMCs from 4 (2 U-CLL and 2 M-CLL) patients alone (**Supplementary Figure 1**) or with pre-activated T cells (**Figure 3**). All animals were sacrificed 5 weeks later, and the numbers of CLL-derived B and T cells in the blood, spleen, and BM evaluated. Busulfan administration did not alter the numbers of CLL B or T cells found at any of the examined sites (**Figure 3A**). Hence, there was not an advantage to busulfan preconditioning NSG recipients when transferring mature CLL B and T cells using the PBMC + aT system.

Additionally, although human hematopoietic cells engraft best in NSG mice after low dose irradiation (11) and this has been successfully employed in transfers of CLL cells (2–4), others (7) and we (not shown) have not found this to enhance xenografting CLL PBMCs.

### Identification of Distinct Temporal Phases of Engraftment and Growth of Chronic Lymphocytic Leukemia B and T Cells In NSG Mice

**Figure 4:**
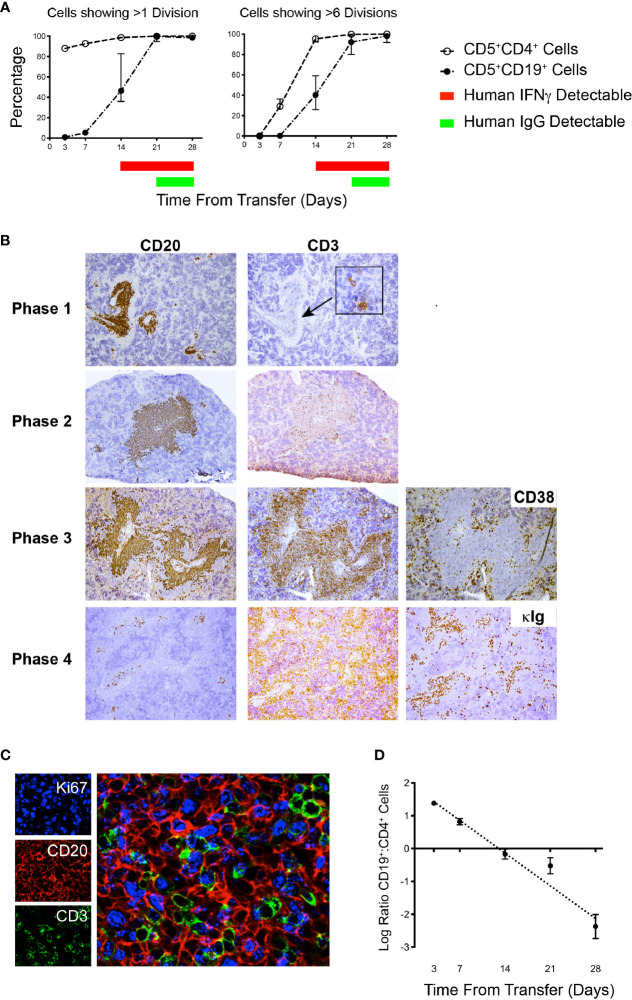
Flow cytometry and IH analyses together with plasma findings over time help define phases of CLL B- and T-cell engraftment and growth. **(A)** Percentage of human CD5^+^CD4^+^ and CD5^+^CD19^+^ spleen-residing cells that have undergone >1 division and >6 divisions as indicated by CFSE dilution (Top) and time of appearance of detectable human IFNγ and human IgG in plasma over 28 days. Data derived from 25 animals using U-CLL1122, with euthanasia performed on 5 animals at each time point. Similar results were obtained using M-CLL1164. **(B)** Representative × 10 original magnification IH of splenic tissue showing typical CD20 and CD3 findings at each phase of engraftment. In Phase 1 the human cells identified are almost exclusively CD20^+^ with virtually no CD3^+^ cells detectable. With progression to Phases 2 and 3, CD3^+^ staining density becomes increased. Beginning in Phase 3 and continuing to Phase 4, CD38^+^ cells (used to identify plasmablasts/plasma cells) appear outside the CD20^+^PVAs. Ultimately, by Phase 4 very few CD20^+^ cells are seen in aggregates, but cytoplasmic Ig^++^ cells are now present (far right hand panel). Representative images obtained from spleens obtained from cases U-CLL1122 (top 2 rows), U-CLL1523 (third row) and U-CLL1083 (bottom row). **(C)** PVAs are strongly Ki67^+^ once both B- and T-cell division occurs. 40x original magnification view using immunofluorescence showing that both CD20^+^ (red) and CD3^+^ (green) cells express Ki67 (blue). Images obtained from U-CLL1301. **(D)** Flow cytometry findings from the experiment in **(A)** indicating the ratio of CD5^+^CD19^+^ cells to CD5^+^CD4^+^ cells.

Since the above indicated that the PBMC + aT approach optimized leukemia-cell growth without a need for preconditioning and that the greatest numbers of cells were found in murine spleens, we categorized the temporal relationships of B- and T-cell expansion in the system. This was done by associating IH studies of tissues and FC analyses of cells from the blood and spleen with the presence of plasma Ig and cytokines over time. This exercise defined 4 distinct, albeit interconnected phases of T- and B-cell growth in NSG recipients. **Table 2** presents the comprehensive analysis after transfers using CLL 1122 and M-CLL 1164; features of the timings of the plasma, FC findings and pertinent findings of IH studies from these and other representative patient samples are shown in **Figure 4**. Of note, since these studies use primary CLL cells, whose biological characteristics vary between patients, the exact timing of each phase between different patient samples is not necessarily temporally identical.

**Table 2:** Phases of engraftment in the PDX model.

Phase	Circulating hCD45^+^ cells	Human Plasma IFNγ	Human Plasma Ig	CFSE pattern of spleen-residing CD4^+^ cells	CFSE pattern of spleen-residing CD5^+^CD19^+^ cells	CD5^+^CD19^+^: CD4^+^ spleen-residing ratio as determined by flow cytometry	Observed ratio of CD20^+^ to CD3^+^ cells seen in splenic tissue by IH	Morphologic description of splenic tissue
Phase 1	Present	Absent	Absent	CFSE dilution patterns show CD4^+^ cells at all stages of division	CFSE dilution patterns show that the majority of cells have undergone <1 division	>1	>1	hCD45^+^ cells localized to perivascular areas, principally CD20^+^. Very low CD3^+^ numbers
Phase 2	Present	Present	Absent	CFSE dilution patterns show >6 division in 90% or more cells	CFSE dilution patterns show CD5^+^CD19^+^ cells at all stages of division	1:1	>1	hCD45^+^ cells localized to perivascular areas, principally CD20^+^, CD3^+^ more obvious
Phase 3	Present	Present	Present	CFSE dilution patterns show >6 division in 90% or more cells	CFSE dilution patterns show >6 division in 90% or more cells	<1	>1	hCD45^+^ present throughout spleen. CD20^+^ cells remain in aggregates intermingled with CD3^+^ cells, red pulp may be infiltrated with CD3^+^ cells and Ig^++^ cells
Phase 4	Present	Present	Present	CFSE shows >6 division in 90% or more cells	Minimal cells	<1	<1	hCD45^+^ throughout spleen. Very few CD20^+^ cells in aggregates, replaced by Ig^++^ cells, red pulp infiltrated with CD3^+^ cells and Ig^++^ cells

Phase 1 is characterized by a predominance of CLL B cells in the peripheral blood and spleen. At this point, leukemic cells are resting, as indicated by the lack of CFSE dilution determined by flow cytometry (3 and 7 days post transfer of cells; **Figure 4A** and **Table 2**). Consistent with this, CLL B-cell numbers in the blood did not significantly change between days 7-21 (**Figure 2**). In addition, transferred cells localizing around blood vessels at this time are virtually all B lymphocytes (as exemplified by CD20 expression), with very few, if any CD3^+^ cells detectable by IH (**Figure 4B**, top panel). Studies using a larger series of CLL cases indicate that these initially leukemic, B-cell restricted perivascular aggregates (PVAs) form between 24 – 72 h after transfer (not shown). The lack of leukemic B-cell division in Phase 1 might result from not yet reaching the numbers of activated T cells needed to provide the requisite levels of human cytokines or the necessary numbers of T-B cell contacts in recipient mice.

Phase 2 is defined by increasing T-cell proliferation and the consequent initiation of substantial CLL B-cell division. This is evidenced by dilution of CFSE intensity in both cell types (T >> B cells) (**Figure 4A** and **Table 2**), increased numbers of CD3^+^ cells as shown by IH (**Figure 4B**, second panel), the presence of Ki67^+^CD20^+^ and Ki67^+^CD3^+^ cells in splenic PVAs (**Figure 4C**), and detection of human IFNγ in the plasma (**Figure 4A** **and** **Table 2**). Note that leukemic B cells outnumber autologous T cells when analyzed by IHC, whereas FC of single cell suspensions prepared from the same spleens reveal a more equal ratio of B and T cells. We speculate this represents the loss of dividing CD20^+^ cells during processing or the inability to mechanically dissociate activated CD20^+^ cells from the tissue (**Figure 4D**); the loss of proliferating B cells upon dissociation of lymphoid tissue is encountered in other settings (12). In this Phase, human Ig is not yet detectable in plasma (**Figure 4A** and **Table 2**).

In Phase 3, a proportion of the spleen-residing CD5^+^CD19^+^ cells have undergone multiple cell divisions (≥ 6) as indicated by complete absence of CFSE as measured by FC, with the degree of CLL B-cell replication varying among patients. In addition, leukemic B cells start to show features of plasmablast/plasma cell differentiation, consistent with the detection of circulating human IgG [**Figure 4A**, 21 days onwards, **Table 2** and (6)]. Importantly, splenic histology continued to show aggregates of CD20^+^ cells, intermingled with CD3^+^ cells as before, with some evidence of plasmablasts/plasma cells at the peripheral margins [as shown by CD38 expression (13)]; this suggested that terminal differentiation of leukemic B cells to CD20^-^ antibody secreting cells was an ongoing process and was not yet complete (**Figure 4B**, third panel).

**Figure 5:**
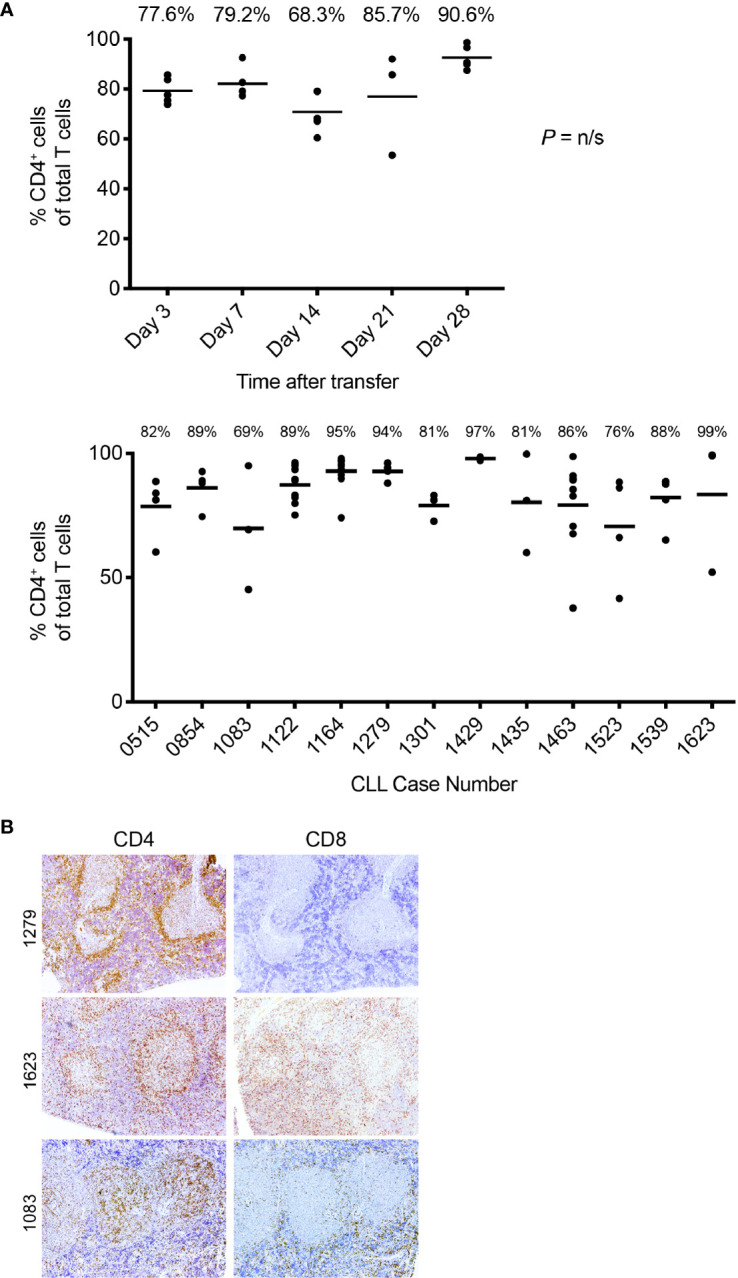
T-cell findings in the PBMC + aT PDX model. **(A)** T cells residing in NSG spleens following transfer are principally CD4^+^. Top. The percentage of CD5^+^CD4^+^as a total of all T cells obtained by flow cytometry analysis of spleen at euthanasia at the time points shown. Data obtained from transfer of cells into 25 mice with euthanasia of 5 animals at each time point. Median percentage CD5^+^CD4^+^ cells indicated by bar and percentage figure. Bottom. The percentage of CD5^+^CD4^+^as a total of all T cells obtained by flow cytometry analysis of spleen at euthanasia. Data obtained from 13 independent experiments (each corresponding to a different CLL case number) where transfer had been made at least 28 days earlier. Median percentage CD5^+^CD4^+^ cells indicated by bar and percentage figure. n/s: no statistically significant differences. **(B)** Representative FC and IH findings of CD4^+^ and CD8^+^ staining in spleen. Images at × 10 original magnification. Pale central areas correspond to CD20^+^PVAs. For CLL1279, CD4^+^ cells are located especially around the rim of the known location of CD20 cells; a minimal number of CD8^+^ cells are present. CLL1623 has both CD4^+^ and CD8^+^ cells; CD4^+^ cells again locate around and within CD20^+^ aggregates. In CLL1083, CD4^+^ cells are densely present within the CD20^+^PVAs with less at the outer margin.

Phase 4 is defined by the virtual complete loss of CD20^+^ cells by IHC, along with an overabundance of cells bearing CD3 and other cells with intense intracellular Ig expression (**Figure 4B**, fourth panel). This is especially the case in animals transferred with higher T:B cell ratios. Serial analyses showed a predominance of CD4^+^ over CD8^+^ T cells with no significant differences in this percentage at all time points (**Figure 5A**, upper). Examining all euthanized animals from 13 different CLL transfers indicated that CD4^+^ cells were the dominant T-cell subpopulation (mean 83.1%); in some animals this was as high as 99% of all T cells (**Figure 5A**, lower). In comparison, based on IHC studies of different CLL transfers, we found that CD4^+^ cells aggregated around and occasionally moved into the PVAs, whereas CD8^+^ cells (when present) showed no particular pattern of localization in splenic tissue (**Figure 5B**).

### Analyses of The Types Of Non-Lymphoid Human Cells and Of Hematopoietic and Non-Hematopoietic Murine Cells in and Around Chronic Lymphocytic Leukemia B Cells Growing in NSG Mice

**Figure 6:**
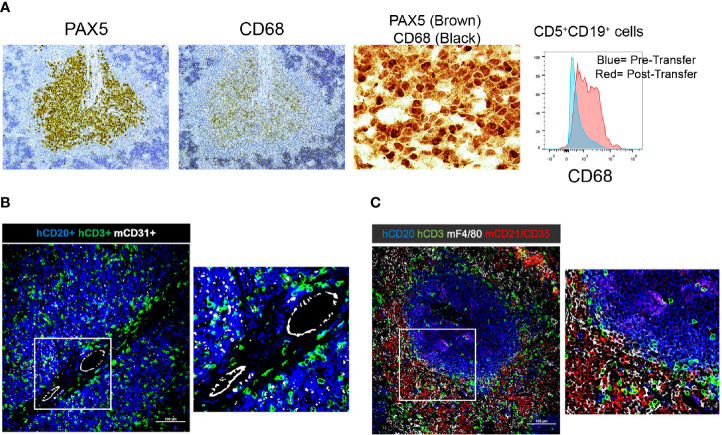
Immunohistochemistry demonstrating the presence and localization of non-B and non-T cells of donor or recipient origin in engrafted spleens. **(A)** 20x (single color PAX5 and CD68) and 60x (dual color) IH images of representative spleens aggregated from 5 independent experiments; results with CLL1279 are demonstrated here. Aggregates contain CD68^+^ cells that are B lymphocytes as indicated by co-localization with PAX5 in dual color staining. Flow cytometry further demonstrates that CD5^+^CD19^+^ cells express CD68 upon transfer into NSG mice. **(B)** 20x and 40x magnification view of human CD20^+^ and CD3^+^ cells and mouse CD31^+^ cells by immunofluorescence of CLL-engrafted spleens; staining from a representative case of at least 10 independent experiments. **(C)** 20x and 40x magnification view of human CD20^+^ and CD3^+^ cells and mouse F4/80^+^ and CD21^+^/35^+^ cells after immunofluorescence staining of the same case.

Next, we examined the presence of other human hematopoietic cells in and around the PVAs; such cells would have been contained in the initial cellular inoculum and have engrafted and persisted in the murine recipients. These searches, however, were fruitless, with no human myeloid cells being found in the peripheral blood or other tissues of recipient mice. This conclusion is based on the absence of cells reactive with CD11b, CD11c, CD33, CD14, and CD15 mAbs identified by FC and IHC (not shown). Notably, we did identify CD68^+^ cells in the spleen by FC and IHC (**Figure 6A**). However, these proved to be human CLL B cells as indicated by co-localization with PAX5^+^ cells by IH and evidence for upregulation of CD68 by CD5^+^CD19^+^ cells by FC following transfer into animals (**Figure 6A**).

Next, we explored the presence and identity of murine non-lymphoid and non-hematopoietic cells and their proximity to human B and T cells. As expected, staining for murine endothelial cells (CD31^+^) confirmed that the CLL cells localized around blood vessels (**Figure 6B**). In addition, we did find murine macrophages (F4/80^+^ cells). However, strikingly, none were located within the CLL B-cell areas; all resided exclusively at the periphery of the PVAs (**Figure 6C**). Similarly, the majority of murine follicular dendritic cells (FDCs; CD21^+^CD35^+^) were located outside the PVAs, although a few were observed within (**Figure 6C**).

### Effects of Route of Administration of Chronic Lymphocytic Leukemia Cells on Engraftment and Growth

**Figure 7:**
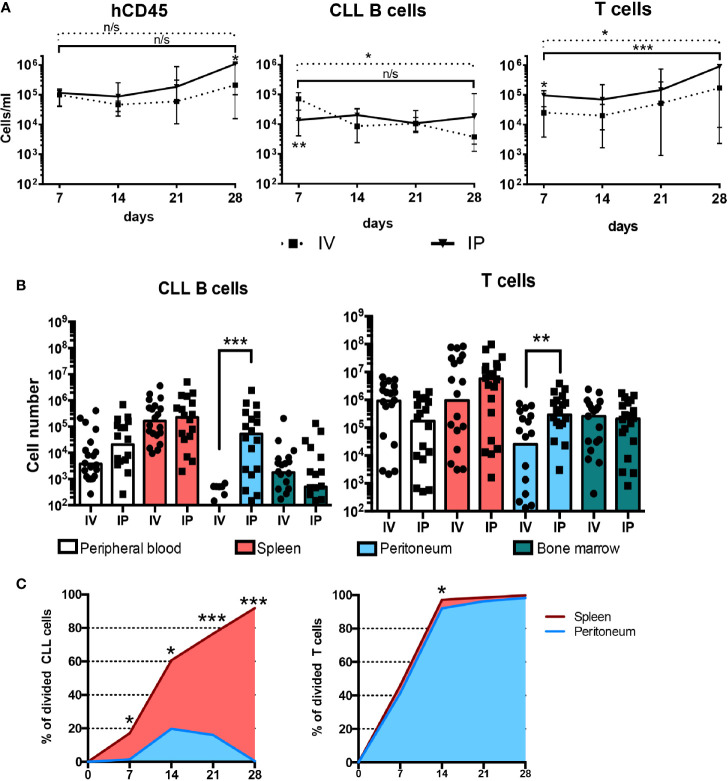
IP administration of CLL B and T cells gives rise to different distributions and activation states. **(A)** Time course quantification of human CD45-expressing cells, CLL B cells and human T cells evaluated by FC in the peripheral blood of mice injected with 20 × 10^6^ CLL PBMCs with 0.5 × 10^6^ activated T cells either iv or ip (4 patients’ samples, 5 mice per patient and condition). Points represent the median and the interquartile range. **(B)** CLL B and T cell absolute counts at day 28 post injection in peripheral blood, spleen, peritoneum and bone marrow from mice injected iv (n = 20) or ip (n = 20). **(C)** Percentage of divided CLL B cells evaluated by CFSE dilution by FC in the spleen and the peritoneum over time. * corresponds to Mann-Whitney U test *P* values < 0.05, **P < 0.01, and ***P < 0.001. n/s: no statistically significant difference.

Finally, we questioned if the route of cell injection—ip vs. iv—affected the level, localization, and expansion of CLL B and T cells in the blood, spleen, BM and peritoneum (**Figure 7**). PBMCs + aT from 4 patients were injected ip or iv, and engraftment and growth followed for 28 days. Of interest, regardless of the route of administration, equal numbers of human CD45^+^ cells were found in the blood at day 7. However, when analyzing CD3^+^ or CD19^+^ cells in the circulation, there were significantly more CLL T cells after iv than ip injection, and more CLL B cells after ip than iv injection (**Figure 7A**). Notably, there were no differences in the numbers of CLL B cells in the spleen, BM, and peripheral blood at day 28 between the types of administration, except for the virtual absence of B cells in the peritoneal cavity if the inoculum was given iv (**Figure 7B**). In contrast, CLL T cells placed initially in the peritoneum were found in greater numbers at day 28 in the spleen than those introduced iv (**Figure 7B**).

When analyzing the populations in the peritoneal cavity and spleen for the extent of cell proliferation, we observed that only a very small fraction of B cells in the peritoneum had divided through day 28 (**Figure 7C**). This was quite different for those CLL B cells that had taken residence in the spleen, regardless of their site of initial transfer, as B lymphocytes at that location divided robustly. The disparity of CLL B-cell division between the peritoneal cavity and the spleen is especially striking in lieu of the high numbers of dividing T cells at both sites (**Figure 7C**).

To assure that these differences would not change if the tissue microenvironments of the NSG recipients were preconditioned, we administered busulfan to the animals 24 h prior to ip injection of CLL B and T cells and then analyzed 5 weeks later the numbers of cells in the spleen, peritoneum and BM in 4 CLL patients (2 M-CLL, and 2 U-CLL) (**Figures 3A, B**). No significant differences in engraftment, localization and proliferation of CLL B cells were found in animals with or without busulfan pre-conditioning. There was, however, a trend for better engraftment of CLL B cells in the spleen and T cells at these sites after preconditioning.

### Utility of the Peripheral Blood Mononuclear Cell + aT Model To Test Therapeutics

**Figure 8:**
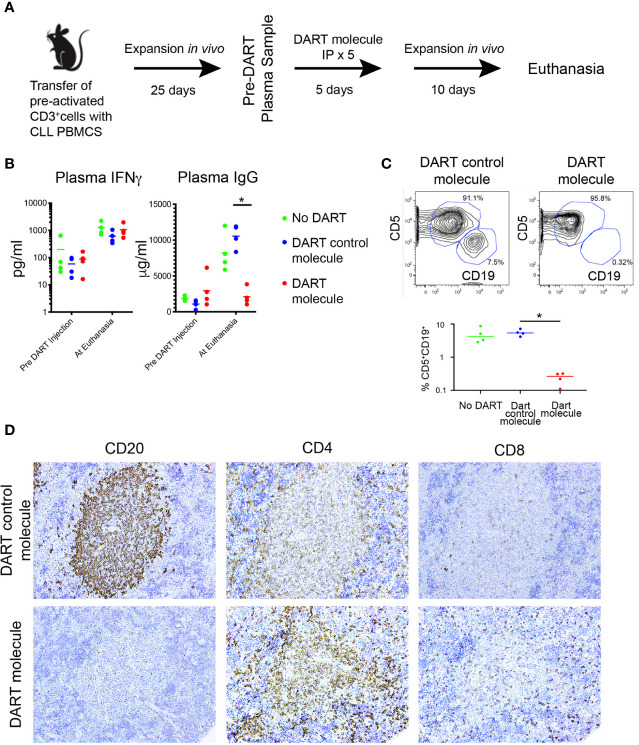
The PBMC + aT PDX model demonstrates the activity of a CD19xTcR-specific DART. **(A)** Mice (n = 15) were injected with 0.5 × 10^6^ anti-CD3/28 + IL-2 pre-activated CLL-derived T cells and 20 × 10^6^ CLL PBMCs on Day 0. Expansion of both T and B cells was determined 25 days post transfer by detection of human IFNγ, and human IgG in murine plasma samples. DART bispecific antibodies (DART molecule, n = 4 animals; or DART control molecule, n = 4 animals) or saline were administered ip from Day 30 to Day 34. Plasma levels of human IFNγ and IgG were further determined at day 46 when euthanasia was performed. Animals with <10 pg/ml IFNγ at Day 25 were excluded from receiving DARTs and were not included in the analysis making 3 groups of 4 animals for each condition. **(B)** Plasma levels of IFNγ and IgG in the 3 groups of animals (n = 12, 4 per group), taken pre-DART injection at Day 25 and at euthanasia at Day 40. Results show no significant differences in IFNγ levels between the 3 groups before DART molecule injection and at euthanasia. In contrast, plasma Ig levels are significantly lower in CD19xTcR DART-treated animals (red dots), compared to the DART control molecule-treated control animals (blue dots). * corresponds to Kruskal-Wallis test *P* value < 0.05. **(C)** FC reveals absence of CD5^+^CD19^+^ cells in DART molecule-treated animals. Illustrative FC plots are representative of hCD45 single cell splenic suspensions obtained at euthanasia. Graph shows median CD5^+^CD19^+^ cells in each group as a percentage of total hCD45^+^ cells isolated. Compared to DART control molecule-treated control animals (blue dots), there are significantly fewer CD5^+^CD19^+^ cells in DART molecule-treated animals (red dots). * corresponds to Kruskal-Wallis test *P* value < 0.05. **(D)** Representative IHC (x20 original magnification) of DART control molecule-treated animals (upper panel) compared to DART molecule-treated animals (lower panel). These results were apparent for both a U-CLL1539 (illustrated in Figure) and M-CLL0545.

To investigate the value of this revised technique in testing the efficacy of therapeutics in a preclinical setting, we used a bispecific retargeted antibody based DART^®^ molecule that engages CD19 on leukemia/lymphoma B cells and the TCR on T lymphocytes (14). This CD19xTCR antibody (hereafter referred to as “DART molecule”) is effective in clearing transplanted lymphoma cell lines co-administered with human PBMCs in a NOD/SCID model and primary patient material from cases of acute lymphoblastic leukemia and diffuse large B cell lymphoma (15). Using the PBMC + aT model, we compared the effects of the DART molecule and of a FITCxTcR antibody that cannot target B cells (referred to as “DART control molecule”) and of saline. **Figure 8A** illustrates the experimental protocol followed.

Each animal in 3 groups (5/group) received iv 20 × 10^6^ CLL B cells with 0.5 × 10^6^ activated autologous T cells from the U-CLL1539 clone. Twenty five days following transfer (Phase 3 of engraftment for this sample), plasma levels of IFNγ and IgG were comparable between the 3 groups of mice (**Figure 8B**, Pre DART Molecule Injection), indicating the comparable nature of CLL B- and T-cell growth among the groups. After receiving the DART molecule, DART control molecule, or saline ip on 5 consecutive days, the animals in each group were euthanized 10 days after the last injection. Plasma levels of IFNγ remained comparable, with the amounts of IFNγ increasing in each group by ~1 log over time (median pre- and post-injection: 70.9 vs. 830.2 pg/ml); this indicated a continued expansion of T cells in all animals regardless of their treatment group (**Figure 8B**).

However plasma IgG levels from the same samples at the same time points from the PBS-treated animals and those receiving the DART control molecule exhibited similar or appreciably higher levels than at the pre-treatment assessment (median: Pre-treatment = 1558 µg/ml; Post-treatment = 9283 µg/ml). This reflected the unchecked growth and differentiation of CLL B cells.

In contrast, plasma IgG levels were significantly lower in the DART molecule-treated animals (**Figure 8B**). In fact, Ig levels in the animals receiving the DART molecule were also lower than those 15 days earlier at the time of initiation of treatment (median: Pre-treatment = 2288 µg/ml, Post-treatment = 1781 µg/ml), further supporting that CLL B-cell numbers were substantially reduced (**Figure 8B**). Consistent with this, flow cytometric analyses of spleen cells from the 3 groups showed that CD5^+^CD19^+^ cells were virtually absent in all animals treated with the DART molecule, whereas those in animals treated with saline or the DART control molecule were sizeable and very similar (**Figure 8C**). Additionally, there were equal numbers of CD5^+^CD4^+^ and CD5^+^CD8^+^ cells in all 3 groups, with the predominant type being CD4^+^; no expansion of the CD8^+^ subset was seen in the DART molecule-treated animals (not shown).

Companion IHC studies indicated the presence of splenic hCD45^+^ cells in all groups, and clearly showed typical CD20^+^ PVAs in untreated (not shown) and DART control molecule-treated animals (**Figure 8D**, upper panel). However, CD20^+^ cells and PVAs were completely absent in 3 of 4 DART molecule-treated animals (**Figure 8D**, lower panel); in the fourth animal, only patchy staining for such cells/structures (estimated as 20% of that seen in untreated animals) was detected. All animals showed dense staining for CD4^+^ cells within PVAs, which was more marked in those animals treated with the DART molecule (**Figure 8D**). CD8^+^ cells did not show any particular localization pattern. Results were replicated in a subsequent experiment using the CLL 0545 clone.

## Discussion

In this study we optimized the engraftment and growth of primary CLL B cells in NSG mice using limited numbers of cryopreserved, primary, peripheral blood patient samples. This was achieved by providing a constant number of polyclonally-activated autologous T cells, pre-stimulated *in vitro*, at the time of transfer into recipient mice.

This approach (PBMCs + aT) has several advantages. First, it increases significantly the percentage of animals that are successfully engrafted when compared to the transfer of PBMCs alone.

Second, the approach eliminates the need to rely on autologous T-cell activation *in vivo*. Moreover, the approach does away with the use of allogeneic antigen-presenting cells (APC) to initiate T-cell activation *in vivo*. As stated previously, the former approach can be suboptimal because the level of histocompatibility disparity between the APC and the CLL cell donor is usually unknown, and hence the extent and degree of CLL T-cell activation occurring in recipient mice differs and is not be predictable nor quantifiable in advance.

Another advantage of the PBMCs + aT approach is the relatively small number of PBMCs needed to carry out the engraftment process (20 × 10^6^). Durig *et al.* have shown that injecting animals, ip and then iv, with larger numbers of PBMCs alone (100 × 10^6^ on each occasion) helps engraftment (1); this is certainly an improvement on a single injection of 100 × 10^6^ PBMCs alone (2, 3, 5, 7). The PBMC + aT method, however, obviates the need for 2 injections and reduces the numbers of cells needed (200 × 10^6^ vs. 20 × 10^6^ cells).

Additionally, all the experiments reported here used cryopreserved patient cells. Although the use of fresh cells can be advantageous (7), this requirement makes answering questions about the biology of CLL cells much more difficult. For example, it would be very cumbersome to compare cells from two types of patients if only fresh cells had to be used, since this would require knowing in advance the existence of the variable to be studied between individual patients and arranging for patients, differing in this variable, to donate the same amount of blood on the same day and at approximately the same time. Furthermore, the use of fresh cells does not allow simultaneous analysis of samples taken at several points in time, making comparisons more restricted and much less rigorous. The capacity to use cryopreserved material removes these and other restrictions.

Finally, the PBMC + aT approach does not require chemical (busulfan) or X-ray preconditioning for effective engraftment of mature CLL cells. This, again, makes the method more convenient and less laborious.

It is important to point out that we assign engraftment as being successful when CLL cells not only survive but also divide in recipient mice. Leukemia-cell survival is a measure of the capacity of CLL cells to accept and benefit from murine microenvironmental signals, actions that a good model should be able to examine. Importantly and additionally, the PBMC + aT method measures the ability of CLL cells to receive and respond to activation signals *in vivo*. Thus, having the transferred cells multiply in recipient animals allows measurement of leukemic-cell birth *in vivo*, a parameter intimately linked to CLL-patient clinical courses (16).

Having a reproducible model for the engraftment and growth of CLL cells *in vivo* allowed us to perform detailed IHC and FC analyses of the kinetics of B-cell proliferation and its relationship with T-cell expansion. We identified 4 phases that a successful transfer traverses. The first phase is characterized by the deposition of quiescent leukemic B cells around blood vessels shortly after cell transfer, hence the term perivascular aggregates (PVAs). During this phase and in Phase 2, activated T cells start and continue to expand at the PVAs, initiating CLL B-cell activation and growth. The majority of T cells are CD4^+^. Anatomically, the few CD8^+^ cells present are interspersed with CLL B cells in the PVAs, whereas the CD4^+^ T cells are more often found at the margins of the PVAs and less so distributed throughout the structures. We have not defined a parameter that associates with the apparent different geographic localization of the CD4^+^cells. By the time of Phase 3, CLL B-cell division is robust, with some cells dividing 6 or more times. Among the latter are cells that phenotypically resemble plasmablasts or plasma cells and secrete CLL Ig. Phase 4 is characterized by the almost complete absence of cells bearing a B-cell phenotype and the predominance of CD4^+^ T cells. In this regard, it is noteworthy that T-cell numbers reach a plateau during Phase 3 and remain constant through Phase 4. The capping of T-cell numbers at a defined level, often at or near day 35 after transfer, is consistent with attaining full occupancy of available lymphoid niches (17). Moreover, the rise in T cell numbers is likely due to the greater rate of proliferation for T cells than B cells and the higher likelihood that T cells recirculate throughout the entire experimental period.

We could not find evidence for human non-lymphoid cells engrafting in this system, indicating that the myeloid and other lineages are inherently not transferrable as mature cells or that the murine microenvironment of NSG mice cannot support their survival and growth. However this finding strongly implies that murine non-B and non-T cells within the NSG microenvironment are capable of providing necessary non-lymphoid support that xenografted CLL cells require; this is compatible with human and murine stromal cells providing supportive cues in *in vitro* (18) and *in vivo* (4). However, it was striking to find that murine macrophages and FDCs, albeit the latter at a somewhat lesser degree were not found within the CLL PVAs, suggesting an active exclusion of these cells from those areas and a lack of their requirement for leukemic-cell proliferation. The reason for this is obscure at this point. However since both of these cell types are APCs (macrophages for T cells and FDCs for B cells), their absence would suggest that the T-B interactions occurring in PVAs are not cognate, but more likely mediated by cytokines (6), and that selection for higher affinity B-cell receptors might not occur, even though B-cell differentiation and BCR diversification along apparent genealogies can occur within these structures (6). The latter findings might reflect selection for non-IG genetic changes, not changes that enhance BCR binding of (auto)antigens.

A full understanding of the kinetics of B- and T-cell engraftment in NSG mice is essential for the analysis of novel therapeutics. We here demonstrate the efficacy of a bispecific antibody (14) whose beneficial effects require the ability to engage autologous T cells in the cytolytic process. As well as being an example of the utility of the PBMC + aT method, these studies highlight and provide an assay whereby the cell interactions between CLL B and T cells and the antibody needed to achieve the therapeutic result can be observed and studied further. Hence, therapeutic agents requiring T-cell participation should take into account that soon after engraftment (end of Phase 1 and beginning of Phase 2) only T cells are dividing, as indicated by the presence of human IFNγ in the blood. Similarly, T-cell numbers are generally limited within PVAs until at least Phase 2. Therefore investigations regarding the utility of agents that exploit T cells (for example, the DART molecule used here) would be expected to have maximal effects only if introduced at or after this phase of engraftment.

Finally, we would like to highlight the biologic insights as well as the technical advantages that our studies of the route of administration of CLL B cells and autologous, activated T cells provide. First, these findings indicate that the (micro)environment that the cells encounter at the time of initial transfer has a substantial effect on migration, cell interactions, and subsequent cell division. Specifically, when activated T and resting CLL B cells are administered iv, the majority of cells leave the blood (B > T) and track to the spleen and BM and not to the peritoneal cavity. In the spleen and BM, both B and T cells undergo vigorous cell division, so much so that CLL B cells are eventually lost, probably for a variety of reasons, including exhaustion because of [1] the numbers of divisions that occur, [2] unsuccessful competition with T cells for survival niches and nutrient cytokines, and [3] elimination T-cell mediated cytolysis.

In contrast when the same cells are deposited in the peritoneal cavity, the vast majority of CLL B cells remaining at that anatomic site are quiescent, despite being among rapidly dividing T cells that one would expect would provide help for B-cell proliferation as occurs when the same cells exit the peritoneum and enter the spleen. Indeed, sufficient numbers of B and T cells migrate to the spleen and BM and undergo robust division at those sites such that B cells achieve the same levels as animals receiving the inoculum ip and in fact T cell numbers exceed that of animals given the same cells iv. So either T cells continue to emerge from the peritoneal cavity into the blood, or they divide in the cavity (or elsewhere) and (re)enter the circulation in higher numbers (**Figure 6B**).

The discrepancies in B-cell division in the peritoneal cavity versus the spleen are reminiscent of normal murine B-1 cells (19) and murine CLL-like TCL1 cells (20). The precise mechanisms responsible for these striking differences in B-cell growth and survival at these anatomic sites are not known, although experiments with murine B-1 cells suggest that soluble factors in the peritoneal cavity might be involved (19).

Regardless, the ability to have quiescent and proliferating subpopulations of human CLL cells in the same animals provides a considerable experimental advantage since this more closely resembles human CLL where there are both dividing and non-dividing pools of leukemic cells (21, 22). In addition, these findings suggest that within this model, the peritoneal cavity resembles the blood and BM of patients, where cells are primarily resting, and the spleen resembles human lymph nodes, where most CLL-cell division occurs (5, 23).

Finally, it is important to recognize that despite the many advantages of this model, the recipient animals do not develop obvious CLL disease. Thus, the model is best used to evaluate the biologic properties of CLL cells and how interactions with autologous T cells modify these properties. Experiments designed to modify the system to model the pathology of CLL are in progress.

In conclusion, we demonstrate here that by using *in vitro* activated autologous T cells in the PBMC + aT PDX model, more reproducible and enhanced engraftment and growth of CLL B cells is achieved using a limited initial inoculum of cryopreserved cells. The system allows comprehensive studies of how CLL B and T cells behave in a xenogeneic setting as well as how these cells interact with and are influenced by their surrounding microenvironment in different tissues and how that might reflect the human leukemic process. Finally, the method provides a robust system for the study of new therapeutic agents.

## Data Availability Statement

The raw data supporting the conclusions of this article will be made available by the authors, without undue reservation.

## Ethics Statement

The studies involving human participants were reviewed and approved by The Institutional Review Board of Northwell Health. The patients/participants provided their written informed consent to participate in this study. The animal study was reviewed and approved by The Institutional Animal Care and Use Committee of Northwell Health.

## Author Contributions

PP and GF conceived the study, performed the experiments, wrote the first draft, and performed the revisions. S-SC conceived the study, performed the experiments, and performed the revisions. JK, KR, SA, and JB provided patient material and reviewed the manuscript. NI and AR performed the experiments and performed the revisions. NC conceived the study, wrote the first draft, and performed the revisions. PP and GF contributed equally to the study. All authors contributed to the article and approved the submitted version.

## Funding

NC received funding for a portion of these studies from Janssen Pharmaceuticals, Inc. NC and KR received philanthropic support from The Nash Family Foundation, the Karches Foundation, The Marks Foundation, and the Jean Walton Fund for Leukemia, Lymphoma, & Myeloma Research. PEMP was funded in part by Blood Cancer UK (Blood Cancer UK grant numbers 11039 and 09011).

## Conflict of Interest

NC received the DART molecule, the DART control molecule, and financial support to carry out the studies in **Figure 8** from Janssen Pharmaceuticals, Inc.

The remaining authors declare that the research was conducted in the absence of any commercial or financial relationships that could be construed as a potential conflict of interest.

## Supplementary Material

Supplementary Table 1 Antibodies for flow cytometry.

Supplementary Table 2 Antibodies used for microscopy studies.

Supplementary Figure 1 Busulfan preconditioning does not provide a clear advantage for xenografting primary CLL cells in the PBMC model. Five NSG mice did not and 5 did receive 25mg/kg busulfan ip 24 h prior to xenografting (10 mice per patient, total 4 patients). Then, 20 × 10^6^ CLL PBMCs were injected iv into NSG mice. Five weeks after cell injection, mice were sacrificed and single cell suspensions from spleen, bone marrow (BM) and peritoneum were analyzed by flow cytometry. Busulfan did not significantly improve the numbers of CLL B cells (top) and T cells (bottom) found at the three anatomic sites. Bar graphs represent the mean fold change (after setting the average cell counts obtained from PBMC mouse spleens as 1); S.E.M. determined by Mann-Whitney U test. n/s: no statistically significant difference.
