# Supplementary material for: A Detailed Analysis of Parameters Supporting the Engraftment and Growth of Chronic Lymphocytic Leukemia Cells in Immune-Deficient Mice
Source: Front Immunol. 2021 Mar 9;12:627020. doi: 10.3389/fimmu.2021.627020 (PMC7985329; doi:10.3389/fimmu.2021.627020)
Supplement: Supplementary Table 1 — Antibodies for flow cytometry. [file Table_1.docx]

**Supplementary Data**

**Table S1: Antibodies for flow cytometry**

| Target Antigen | Isotype | Clone | Company |  | Fluorochrome |
| --- | --- | --- | --- | --- | --- |
| CD4 | IgG1 | RPA-T4 | BD Biosciences |  | PE |
| CD4 | IgG1 | RPA-T4 | BD Biosciences |  | APC-H7 |
| CD5 | IgG1 | UCHT2 | BioLegend |  | PE-Cy7 |
| CD8 | IgG1 | RPA-T8 | BD Biosciences |  | V450 |
| CD8 | IgG1 | SK1 | BD Biosciences |  | APC-H7 |
| CD8 | IgG1 | SK1 | BD Biosciences |  | APC |
| CD19 | IgG1 | HIB19 | BD Biosciences |  | APC |
| CD19 | IgG1 | J3-119 | Beckman Coulter |  | ECD |
| CD45 (human) | IgG1 | H130 | Invitrogen |  | PO |
| CD45 (mouse) | IgG2b (Rat) | 30-F11 | BD Biosciences |  | PerCP |
| CD45 (mouse) | IgG2b (Rat) | 30-F11 | BD Biosciences |  | PE-Cy7 |
| CD45 RA | IgG2b | H1100 | BD Biosciences |  | PE-Cy7 |
| CD68 | IgM | NK-1 | BD Biosciences |  | APC |
|  |  |  |  |  |  |

All are murine monoclonal antibodies unless otherwise stated.

All markers were analyzed for surface membrane staining with the exception of anti-CD68.
