# Supplementary material for: A Detailed Analysis of Parameters Supporting the Engraftment and Growth of Chronic Lymphocytic Leukemia Cells in Immune-Deficient Mice
Source: Front Immunol. 2021 Mar 9;12:627020. doi: 10.3389/fimmu.2021.627020 (PMC7985329; doi:10.3389/fimmu.2021.627020)
Supplement: Supplementary Table 2 — Antibodies used for microscopy studies. [file Table_2.docx]

**Table S2: Antibodies used for microscopy studies**

| Target Antigen | Species | Isotype | Clone | Company |
| --- | --- | --- | --- | --- |
| hCD3 | Rat | IgG1 | CD3-12 | AbD Serotec |
| hCD4 | Mouse | IgG1 | 4B12 | Leica |
| hCD8 | Mouse | IgG1 | 1A5 | Leica |
| hCD20 | Mouse | IgG2a | L26 | Dako |
| hCD68 | Mouse | IgG1 | KP1 | Dako |
| hIgλ | Rabbit | Polyclonal |  | Dako |
| hIgκ | Rabbit | Polyclonal |  | Dako |
| hKi67 | Mouse | IgG1 | MIB-1 | Dako |
| hPAX5 | Mouse | IgG1 | DAK-Pax5 | Abcam |
| mF4/80 | rabbit | IgG1 | D2S9R | Cell Signaling |
| mCD31 | rabbit | Polyclonal |  | Abcam |
| mCD21/CD35 | Rat | IgG2 | 7E9 | Biolegend |
