# Supplementary figures and images for: A Detailed Analysis of Parameters Supporting the Engraftment and Growth of Chronic Lymphocytic Leukemia Cells in Immune-Deficient Mice
Source: Front Immunol. 2021 Mar 9;12:627020. doi: 10.3389/fimmu.2021.627020 (PMC7985329; doi:10.3389/fimmu.2021.627020)

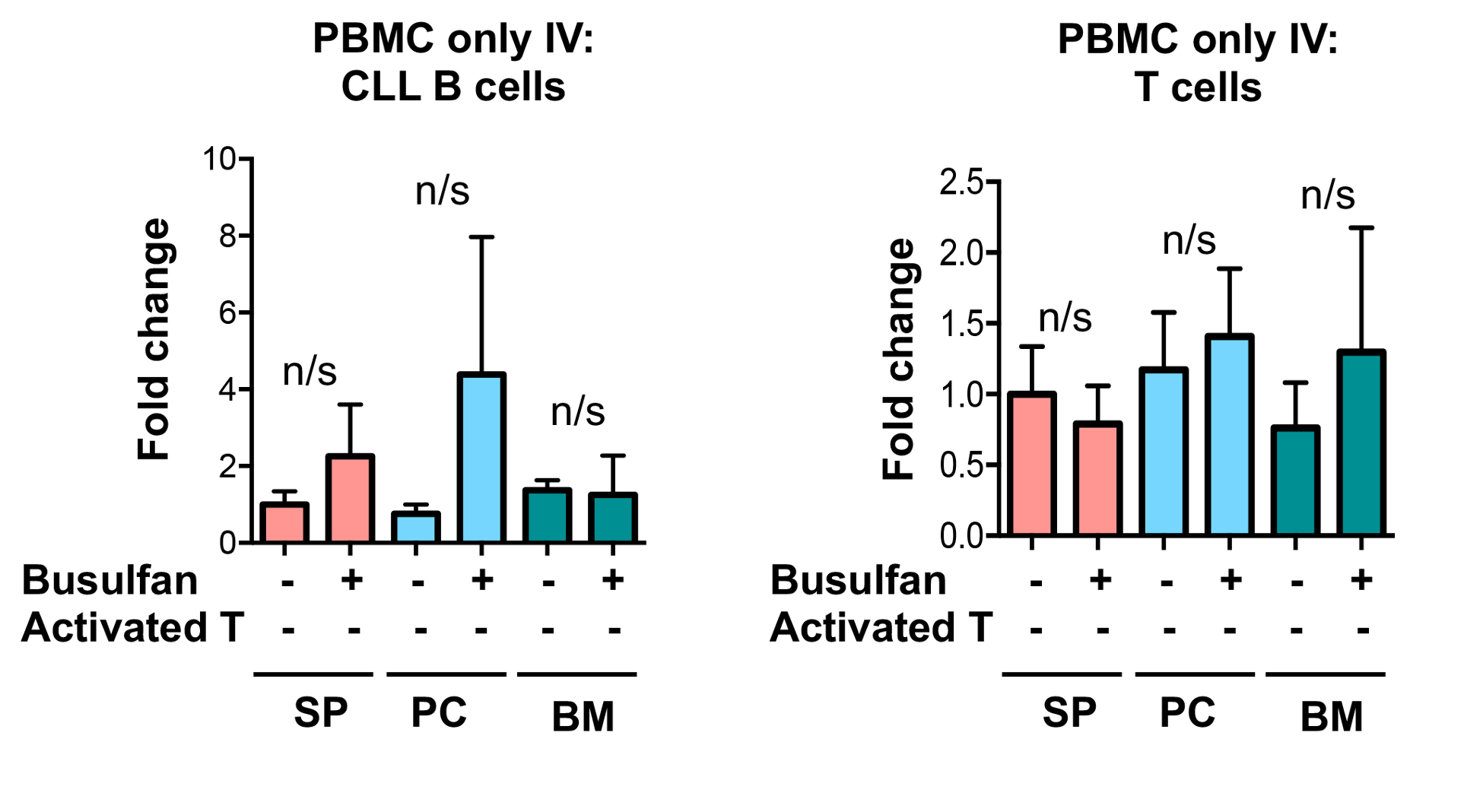

Supplement: Supplementary Figure 1 — Busulfan preconditioning does not provide a clear advantage for xenografting primary CLL cells in the PBMC model. Five NSG mice did not and 5 did receive 25mg/kg busulfan ip 24 h prior to xenografting (10 mice per patient, total 4 patients). Then, 20 × 106 CLL PBMCs were injected iv into NSG mice. Five weeks after cell injection, mice were sacrificed and single cell suspensions from spleen, bone marrow (BM) and peritoneum were analyzed by flow cytometry. Busulfan did not significantly improve the numbers of CLL B cells (top) and T cells (bottom) found at the three anatomic sites. Bar graphs represent the mean fold change (after setting the average cell counts obtained from PBMC mouse spleens as 1); S.E.M. determined by Mann-Whitney U test. n/s: no statistically significant difference. [file Image_1.jpeg]
